# Allergy to hamster - 4 case reports

**DOI:** 10.1186/2045-7022-4-S2-P40

**Published:** 2014-03-17

**Authors:** Christiane Hilger, P Ved Dubey, Delphine Lentz, Martine Morisset, Christiane Lehners, K De Prabir, François Hentges

**Affiliations:** 1 CRP-Santé, Infection & Immunity, Luxembourg, Luxembourg; 2 Centre for Cellular & Molecular Biology, Mol Endocrinology, Biochemistry & Mol Biology, Hyderabad, India; 3 Centre Hospitalier de Luxembourg, National Unit of Allergology, Luxembourg, Luxembourg

## Background

Hamsters are increasingly popular as domestic pets. Different species can be found in the domestic setting: the Syrian (golden) hamster (*Mesocricetus auratus*), the European hamster (*Cricetus cricetus*), the Siberian hamster (*Phodopus sungorus*) and the Rhoborovski hamster (*Phodopus roborovskii*). Several cases of asthma upon contact with hamsters and anaphylaxis following hamster bites have been described, but the allergen(s) responsible are either unidentified or poorly characterized. In the Syrian hamster, salivary lipocalins were found to be specifically expressed in male submandibular glands. MSP (male specific submandibular salivary gland protein) is detectable in saliva as two major forms of 20.5 and 24 kDa. The objectives of the present study were to determine if MSP is an allergen, to analyse IgE reactive proteins in different hamster fur extracts and to determine patient reactivity to different extracts.

## Methods

Hamster protein hair extracts were prepared from four different hamster species. Hamster-allergic patients were selected based on their clinical history and a positive IgE test to hamster epithelium. IgE reactive proteins were analysed by immunoblot. Recombinant MSP was expressed in *E. coli*. Natural MSP was purified from Syrian hamster and MSP anti-serum was raised in rabbits.

## Results

Three of the patient sera (P2, P3, P4) had IgE antibodies against proteins of 20.5 and 24 kDa of Syrian hamster fur extract. These proteins could be identified as MSP. Similar bands were visible in the extract of the European hamster. Two patients (P1, P2) showed IgE reactive bands at 17-21.5 kDa in Siberian and Rhoborovski hamster extract. P1 and P2 were further characterized by basophil release assay. They reacted positively with both Phodopus species, but negative with Syrian and European hamster. Both patients however reacted with fur extract of all 4 species in skin prick test.

## Conclusion

The salivary lipocalin MSP was identified as allergen of the Syrian hamster. Cross-reactive proteins were detected in the European hamster. Major allergens of Siberian and Roborovski hamster seem to be different from those identified in the Syrian hamster.

